# Fucoidan: Structure and Bioactivity

**DOI:** 10.3390/molecules13081671

**Published:** 2008-08-12

**Authors:** Bo Li, Fei Lu, Xinjun Wei, Ruixiang Zhao

**Affiliations:** School of Food Science, Henan Institute of Science and Technology, Xinxiang 453003, Henan, P.R. China; E-mails: libohnxx@163.com (Fei Lu), wxj@hist.edu.cn (Xinjun Wei), zrx338@163.com (Ruixiang Zhao)

**Keywords:** Fucoidan, structure, bioactivity

## Abstract

Fucoidan refers to a type of polysaccharide which contains substantial percentages of l-fucose and sulfate ester groups, mainly derived from brown seaweed. For the past decade fucoidan has been extensively studied due to its numerous interesting biological activities. Recently the search for new drugs has raised interest in fucoidans. In the past few years, several fucoidans’ structures have been solved, and many aspects of their biological activity have been elucidated. This review summarizes the research progress on the structure and bioactivity of fucoidan and the relationships between structure and bioactivity.

## Introduction

Fucoidans, polysaccharides containing substantial percentages of l-fucose and sulfate ester groups, are constituents of brown seaweed and some marine invertebrates (such as sea urchins and sea cucumbers) [1,2]. The polysaccharide was named as “fucoidin” when it was first isolated from marine brown algae by Kylin in 1913. Now it is named as “fucoidan” according to IUPAC rules, but some also called it fucan, fucosan or sulfated fucan. 

For the past decade fucoidans isolated from different species have been extensively studied due to their varied biological activities, including anticoagulant and antithrombotic, antivirus, antitumor and immunomodulatory, anti-inflammatory, blood lipids reducing, antioxidant and anticomplementary properties, activitiy against hepatopathy, uropathy and renalpathy, gastric protective effects and therapeutic potential in surgery. Compared with other sulfated polysaccharides, fucoidans are widely available from various kinds of cheap sources, so more and more fucoidans have been investigated in recent years to develop the drugs or functional foods. This paper summarizes the research progress on structure and bioactivity of fucoidans isolated from brown seaweeds and the relationships between their structures and bioactivity.

## Structure

Since Kylin firstly isolated fucoidan in 1913, the structures of fucoidans from different brown seaweeds have been investigated. Fucoidans from several species of brown seaweed, for example *Fucus vesiculosus*, have simple chemical compositions, mainly being composed of fucose and sulfate. But the chemical compositions of most fucoidans are complex. Besides fucose and sulfate, they also contain other monosaccharides (mannose, galactose, glucose, xylose, etc.) and uronic acids, even acetyl groups and protein. Furthermore, the structures of fucoidans from different brown algae vary from species to species. Despite all that, some fucoidans’ structures or their structural backbones have been elucidated.

### Fucoidans mainly composed of fucose and sulfate

Fucoidan prepared from *Fucus vesiculosus* is commercially available at present. It is composed of 44.1% fucose, 26.3% sulfate and 31.1% ash, plus a little aminoglucose; its [α]_D_ is -123° [3,4]. On the basis of the results of methylation and alkali treatment, Conchie and O’Neill found the main component unit was 1,2-α-fucose and most of sulfate groups were located at position C-4 of the fucose units [5,6]. Anno *et al.* isolated l-fucose 4-sulfate from it and the IR spectrum suggested that the sulfate group was substituted at the axial C-4 position of the l-fucospynanose [7].

**Figure 1:**
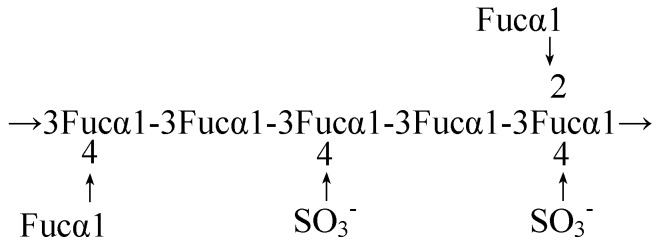
Pankter model for the average structure of fucoidan.

The structural model of fucoidan of *F. vesiculosus* suggested by Conchie was accepted for forty years. In 1993, on the GC/MS data of methylation Pankter *et al* revised this structural model suggesting that the core region of fucoidan was primarily a polymer of α-(1→3) linked fucose with sulfate groups substituted at the C-4 position on some of the fucose residues; fucose was also attached to this polymer to form branched points, one for every 2-3 fucose residues within the chain (Figure 1). Pankter also explained the possible reasons for the different observations of Conchie. First was the difference of preparation method: fucoidan analyzed in Conchie’s studies was extracted with hot water, rather than acid extraction used by Pankter, which has been the basis of the commercial preparation in recent years; secondly, their methylation methods were different; finally, Conchie analyzed the structure according to the chemical and chromatographic properties of the methylated products, and Pankter confirmed the methylated products by GC-EIMS [8]. 

Bilan *et al* reported that fucoidans from the brown seaweeds *F. evanescens* C. Ag, *F. distichus* and *F. serratus* L. were consisted of fucose, sulfate and acetate [2,9,10]. Fucoidan of *F. evanescens* C. Ag. has a linear backbone of alternating 3- and 4-linked α-l-fucopyranose 2-sulfate residues: →3)-α-l-Fuc*p*(2SO_3_^-^)-(1→4)-α-l-Fuc*p*(2SO_3_^-^)-(1→, with additional sulfate occupying position 4 in a part of 3-linked fucose residues, whereas a part of the remaining hydroxyl groups was randomly acetylated [2]. Fucoidan of *F. distichus* is built up of disaccharide repeating units: →3)-α-l-Fuc*p*-(2,4-di-SO_3_^−^)-(1→4)-α-l-Fuc*p*-(2SO_3_^−^)-(1→. The regular structure may be only slightly masked by random acetylation and undersulfation of several disaccharide repeating units [9]. Fucoidan from *F. serratus* l. has a branched structure, its backbone is →3)-α-l-Fuc*p*-(1→4)- α-l-Fuc*p*-(1→, about half of the 3-linked residues are substituted at C-4 by α-l-Fuc*p*-(1→4)- α-l-Fuc*p*-(1→3)-α-l-Fuc*p*-(1→trifucoside units. Sulfate groups occupy mainly C-2 and sometimes C-4, although 3,4-diglycosylated and some terminal fucose residues may be nonsulfated. Acetate groups occupy C-4 of 3-linked Fuc and C-3 of 4-linked Fuc in a ratio of about 7:3. The fucoidan also contains small amounts of xylose and galactose [10]. A sulfated fucan from *Stoechospermum marginatum* has a backbone of (1→4)- and (1→3)-linked-α-l-fucopyranosyl residues that are substituted at C-2 and C-3, and that fucosyl residues are sulfated mostly at C-2 and/or C-4 [11].

The ultrastructure of fucoidan can be studied using a variety of electron microscopy techniques. Sulfated fucan from *Padina gymnospora* forms well-organized ultrastructures and exhibits particles with polygonal forms with a polycrystalline structure. These particles are in fact constituted by sulfated fucan molecules since they are recognized by a lectin specific for α-l-fucosyl residues. X-ray microanalysis reveal that S is a constituent element, as expected for sulfated groups [12].

### Fucoidans from other brown seaweeds

The chemical composition of fucoidan from *F. vesiculosus* is relatively simpler, but most of fucoidans have a complex composition. In 1962 Schweiger isolated a polysaccharide from *Macrocytis pyrifera* and the ratio of fucose to galactose was 18:1, then he first reported that fucoidan was not a pure fucan sulfate but the heteropolymer of fucose, galactose and trace xylose [13]. Henceforth, other sugars such as mannose, glucose, xylose and glucuronic acid (GlcA) had been found in fucoidans from different brown seaweeds (see Table 1), which increased the difficulty of structural analysis. 

The structural character of 1→3 linked fucopyranose is presented in fucoidans of *Ecklonia kurome* and *Chorda filum* [1,14]. A fucoidan fraction isolated from *E. kurome* has a highly branched structure, its backbone is (1→3)-l-fucosyl and sulfate groups mainly attach to C-4 [14]. Fucoidan isolated from *Chorda filum* contains a poly-α-(1→3)-fucopyranoside backbone with a high degree of branching, mainly of α-(1→2)-fucopyranoside single units. Some fucopyranose residues are sulfated at *O*-4 (mainly) and *O*-2 positions. Some α-(1→3)-fucose residues are shown by NMR to be 2-*O*-acetylated [1].

Nevertheless, 1→2 or 1→4 linked fucopyranose can also be found in some brown seaweeds. Fucoidans of *Himanthalia lorea* and *Bifurcaria bifurcata* have (1→2)- and (1→3)-linked fucose residues with sulfation at C-4. The GlcA and xylose residues are 1→4 linked and not sulfated, they are on the periphery of highly branched molecules [15]. GlcA, mannose and glucose in fucoidan of *Padina pavonia* are also 1→4 linked and fucose was 1→2 linked [16,17]. Chevolot *et al.* reported that the predominant repeating structure of fucoidan from *Ascophyllum nodosum* was [→3)-α-l-Fuc(2SO_3_^-^)-(1→4)-α-l-Fuc(2,3diSO_3_^-^)-(1]_n_ [18]. Marais *et al.* reported that a fucoidan fraction purified from *A. nodosum* consisted of a highly branched core region with primarily α-(1→3)-fucosyl residues and a few α-(1→4)-linkages. Branch points were at position 2 of the →3)-fucosyl internal residues, while sulfate groups were at positions 2 and/or 4 [19].

**Table 1:** Chemical compositions of some fucoidans.

Brown seaweed	Chemical composition
*F. vesiculosus* [3,4]	fucose, sulfate
*F. evanescens* C.Ag. [2]	fucose/sulfate/acetate (1/1.23/0.36)
*F. distichus* [9]	fucose/sulfate/acetate (1/1.21/0.08)
*F. serratus* L. [10]	fucose/sulfate/acetate (1/1/0.1)
*Lessonia vadosa* [20]	fucose/sulfate (1/1.12)
*Macrocytis pyrifera* [3]	fucose/galactose (18/1), sulfate
*Pelvetia wrightii* [21]	fucose/galactose (10/1), sulfate
*Undaria pinnatifida* (Mekabu) [22]	fucose/galactose (1/1.1), sulfate
*Ascophyllum nodosum* [23]	fucose(49%), xylose(10%), GlcA(11%), sulfate
*Himanthalia lorea and Bifurcaria bifurcate* [15]	fucose, xylose, GlcA, sulfate
*Padina pavonia* [16,17]	fucose, xylose, mannose, glucose, galactose, sulfate
*Laminaria angustata* [24]	fucose/galactose/sulfate (9/1/9)
*Ecklonia kurome* [25]	fucose, galactose, mannose, xylose, GlcA, sulfate
*Sargassum stenophyllum* [26]	fucose, galactose, mannose, GlcA, glucose, xylose, sulfate
*Adenocytis utricularis* [27]	fucose, galactose, mannose, sulfate
*Hizikia fusiforme* [28]	fucose, galactose, mannose, xylose, GlcA, sulfate
*Dictyota menstrualis* [29]	fucose/xylose/uronic acid/galactose/sulfate (1/0.8/0.7/0.8/0.4) and (1/0.3/0.4/1.5/1.3)
*Spatoglossum schroederi* [30]	fucose/xylose/galactose/sulfate (1/0.5/2/2)

**Figure 2:**
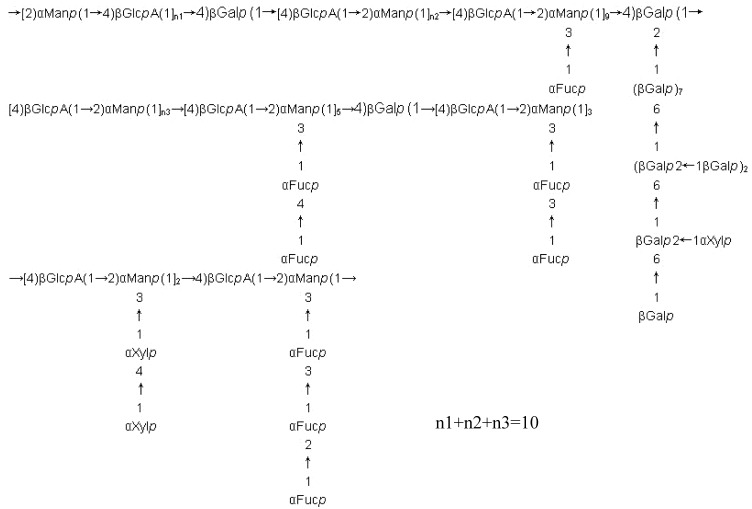
Presumptive structure of fucoidan fraction F32 from *Hizikia fusiforme*.

A fucoidan fraction F32 containing a fucose-free core was isolated from *Hizikia fusiforme*. The sugar composition of F32 was mainly fucose, galactose, mannose, xylose and GlcA, sulfate was 21.8%, and Mw was 92.7 kDa. Its structural core was composed of →2)-α-d-Man(1→ and →4)-β-d-GlcA(1→ alternately, while a little →4)-β-d-Gal(1→ was mixed in them (see Figure 2). Sulfate groups were at C-6 of →2,3)Man(1→, C-4 and C-6 of →2)Man(1→, C-3 of →6)Gal(1→, C-2, C-3 or C-4 of fucose, while some fucose had two sulfate groups. There was no sulfate group on GlcA and xylose. There was 1.2% protein in F32, and the reducing ends of the sugars linked mainly with Thr, and little with Ser through O-glycosidic bonds [28].

The highly sulfated oligosaccharides obtained by partial hydrolysis of fucoidan can be analyzed by ESI-MS, which gives additional information about the structure of this highly complex polysaccharide. [31, 32]. 

### Position of sulfate groups

The position of sulfate groups is important to the biological activities of sulfated polysaccharides. The methods of determining the sulfate position include infrared specvtroscopy, desulfation, stability of sulfate esters to alkali and methylation analysis, etc.

Zvyagintseva *et al.* analyzed the water-soluble polysaccharides of the mostly distributed Far-Eastern brown algae (*L. cichorioides, F. evanescens* and *L. japonica*). The IR spectra of fucoidans showed that most sulfate groups were in axial positions and the remainder were in equatorial positions according to a strong band at 842 cm^-1^ and a shoulder at 820 cm^-1^ in the spectra [33]. Infrared is the generally used method for determining the sulfate position. Nevertheless, erroneous conclusions can be obtained if the position of sulfate group is determined only by the infrared spectrum, because besides the C-O-S vibration in 820-850 cm^-1^ region, there was also C-H bending vibration of sugar reducing end, which affected the judgment on the position of sulfate group. So it is necessary to compare the infrared spectroscopy results with the stability of sulfate esters to alkali and methylation analysis to determine the sulfate position [34].

Mass spectrometry and NMR also can be used to determining the sulfate position. Tissot *et al.* analyzed three isomers 2-*O*-, 3-*O*-, and 4-*O*-sulfated fucose using electrospray ionization trap mass spectrometry (ESI-MS) and capillary electrophoresis. The results showed that it was possible to differentiate between these three positional isomers of sulfated fucose based on their fragmentation pattern upon MS/MS experiments [35]. Grachev *et al.* studied the influence of sulfate groups at C-2 and C-4 on the conformational behavior of fucoidan fragments with homo-(1→3)-linked backbone by NMR. It was shown that *O*-sulfation of (1→3)-linked oligofucosides restricts their conformational flexibility and changes the conformational equilibrium if compared with the parent nonsulfated oligosaccharides [36].

### Fucosidase

Specific enzymatic methods can be used to simplify the structure of fucoidan and reduce the difficulty of analytical works. Enzymes capable of degrading fucoidans have been isolated from several marine species.

A fucosidase extracted from digestive glands of the marine mollusc *Pecten maximus* was shown to possess fucoidan-degrading activity. This activity was able to release l-fucose from fucoidan of *A. nodosum*, and markedly reduce the molecular size of the polysaccharide. An enzymatically degraded fucoidan provided new insight into the structure of the polysaccharide. The fucoidan has a randomly organized structure, involving (1→3)- and (1→4)-linked unsulfated and 2-sulfated-α-l-fucose residues [37,38]. 

Kusaykin *et al.* compared the specificities of fucoidanases from the marine microorganisms *Pseudoalteromonas citrea* KMM 3296 and the marine mollusk *Littorina kurila*. The enzymes possess similar specificities and catalyze the cleavage of accessible α-(1→3)-fucoside bonds in fucoidans with highly sulfated α-(1→4; 1→3)-l-fucooligosaccharides. A high degree of sulfation of the fucose residues in fucoidans makes α-(1→3)-l-fucoside bonds inaccessible for the action of the studied enzymes. The maximum degree of cleavage of fucoidan was achieved by the fucoidanase from the marine bacterium [39].

### Complexity of fucoidan structures

Many studies show that the ordered fucoidans may contain a linear backbone built up of (1→3)-α-l-Fuc or alternating (1→3)-α-l-Fuc and (1→4)-α-l-Fuc, (1→2)-α-l-Fuc sometimes being present in the backbone branching. Sulfate groups often occupy the *C*-2 or/and *C*-3, *C*-4 of fucose. For fucoidans containing a lot of uronic acid (UA) and hexose, the structural core may be built of alternating UA-hexose, because this structure is very stable, and other sugars present in the branching of core, just like the structure of Figure 2. However, a central core consisting mainly of 3-β-D-GlcA-1→ or 4-β-D-GlcA-1→ was found in fucoidan of *Padina gymnospora*. [40]. Fucoidans' structures includes sugar composition, sulfate content and position, molecular weight, linkage mode and sequence of sugar residues, etc. Sulfate groups would disturb the accuracy of methylation analysis and linkage type of uronic acid residues can not be analyzed in GC/MS, so desulfation and carboxyreduction are needed in structural investigation. Moreover, for fucoidans of large molecular weight, partial hydrolysis is also needed in NMR analysis. The above pretreatments can simplify the fucoidan’s structure, but also bring some uncertainties caused by these treatments.

Large numbers of structural studies show that the chemical compositions and structures of fucoidans from brown algae are very complex and their structures vary from species to species. The difference in backbone structures of fucoidans likely reflects the fundamental difference in fucoidans biosynthesis. Despite numerous structural studies of algal fucoidans, their fine structure remains unclear due to the absence of strict regularity, the presence of numerous minor components in some of them (pentose, hexsoe, uronic acids, and sometimes protein component) and random sulfation and acetylation. Sulfated fucan isolated from echinoderms have usually linear backbones and regular sulfation patterns resulting in the formation of oligosaccharide repeating units. The structures of these repeating units can be determined unambiguously, especially by using high-field NMR spectroscopy, and hence, correlation between structures and biological action of polysaccharides may be outlined [41,42,43,44,45]. Unfortunately, the structures of algal fucoidans are much more complicated. The algal polysaccharides are usually heterogeneous and branched, usually only partial information on their structures can be obtained by NMR spectroscopy. Controversial data may be found in the literature, even about the structure of the most carefully studied fucoidan from *F. vesiculosus*.

The same specific brown seaweed possibly possesses different structural fucoidans. Duarte *et al.* reported that *Sargassum stenophyllum* biosynthesized two different sets of fucoidans. One of them is characterized by higher percentages of GlcA and fewer sulfate groups, which are situated on different sugar units, fucose was the major component but other sugars like galactose, mannose, GlcA, glucose and xylose were also in substantial amounts. Another fucoidan contains small amounts of GlcA and high percentages of sulfate groups, which are concentrated on the fucose residues, with only fucose and galactose as major components. Moreover the general basic structure of one fucoidan has a formal resemblance to that of the fucosylated chondroitin sulfates from the body wall of sea cucumbers, namely, a linear core (formed by (1→6)-β-d-Gal and/or (1→2)-β-d-Man units) with branched chains of “fucan” (formed by (1→3) and/or (1→4)-α-l-Fuc, (1→4)-α-d-GlcA, teminal β-d-Xyl and, sometimes, (1→4)-α-d-Glu) [26].

Fucoidans extracted by different methods may also have different structures. Ponce *et al.* reported that fucoidan of *Adenocytis utricularis* extracted at room temperature was composed of mainly fucose, galactose and sulfate ester (the “galactofucan”). The fucoidan extracted at 70°C was composed mainly of fucose, accompanied by other monosaccharides (mostly mannose, but also glucose, xylose, rhamnose and galactose), significant amounts of uronic acids and low proportions of sulfate ester, namely “uronofucoidan” [27].

## Bioactivity

### Anticoagulant and antithrombotic activity

Fucoidans have a wide variety of biological activities, but their potent anticoagulant action is by far the most widely studied. Nishino *et al.* tested anticoagulant activities of fucoidans isolated from nine brown seaweed species, including activated partial thromboplastin time (APTT), thromboplastin time (TT) and anti-factor Xa activity in comparison with values of heparin (167 units/mg). All fucoidans showed some TT (0-35 units/mg) and APTT (12-38 units/mg) activities, whereas anti-factor Xa activity was not remarkable in any fucoidans. Among the fucoidans tested, that of *E. kurome* exhibited the highest activity with respect to APTT (38 units/mg) and TT (35 units/mg), APTT and TT of *H. fusiforme* were 25 units/mg and 22 units/mg respectively [46]. The anti-thrombin activity of fraction F-4 of fucoidan from *L. angustata* var. *longissima* is 200 units/mg, as compared with heparin (140 units/mg) [24].

Cumashi *et al.* studied the anticoagulant properties of fucoidans obtained from nine species of brown algae. All fucoidans, except that from *Cladosiphon okamuranus,* carrying substantial levels of 2-*O*-α-d-glucuronopyranosyl branches in the linear (1→3)-linked poly-α-fucopyranoside chain, exhibited anticoagulant activity as measured by APTT, whereas only fucoidans from *L. saccharina*, *L. digitata*, *F. serratus*, *F. distichus*, and *F. evanescens* displayed strong antithrombin activity in a platelet aggregation test [47].

Many studies showed that the anticoagulant activity of fucoidan maybe have some relation with sulfate content and position, molecular weight and sugar composition. The higher content of sulfate groups often presents the higher anticoagulant activity in native fucoidans (*E. kurome*, *H. fusiforme*, etc.). However, the increment of the anticoagulant and the antithrombin effects gradually decreased with increase in the sulfate content of the fucans [25, 48]. Oversulfated fucoidan prepared from chemical sulfation of native fucoidan also supports it. Nishino *et al.* prepared three species of oversulfated fucans having different sulfate contents (sulfate/sugar ratio, 1.38–1.98) by chemical sulfation of a fucan sulfate (sulfate/sugar ratio, 1.28) isolated from *E. kurome*. The respective activities (for APTT and TT) of the oversulfated fucans increased to 110–119% and 108–140% of the original values increased in their sulfate content. The anticoagulant activity with respect to APTT (173 units/mg) of an oversulfated fucan (sulfate/sugar ratio, 1.98) was higher than that (167 units/mg) of heparin used as a standard. The heparin cofactor II-mediated antithrombin activity of the oversulfated fucans also increased significantly with increase in sulfate content [49]. Qiu *et al.* reported that the oversulfated fucoidan showed four times higher anticoagulant activity in doubling prothrombin time of normal citrated human plasma in comparison with native fucoidan [50].

The position of sulfate groups on sugar residues is also very important for the anticoagulant activity of fucoidan. The activity relates to the concentrations of *C*-2 sulfate and *C*-2,3 disulfate [51], moreover 2,3-disulfated sugar residue is the common structural feature in anticoagulant fucoidans [18,52]. Duarte *et al.* reported that the anticoagulant properties of fucoidans were mainly determined by the fucose sulfated chains, specially by the disulfated fucosyl units [26]. Silva *et al*. reported that 3-*O*-sulfation at C-3 of 4-α-l-fucose-1→ units was responsible for the anticoagulant activity of fucoidan from *Padina gymnospora* [40].

Fucoidan requires an enough long sugar-chain and a comfortable conformation to bind the thrombin, so a certain large molecular weight is needed to achieve anticoagulant activity. The native fucoidan (MW 320,000) from *Lessonia vadosa* (Phaeophyta) showed good anticoagulant activity, whereas the radical depolymerized fraction (MW 32,000) presented weak anticoagulant activity [20]. A slight decrease in the molecular size of the sulfated fucan dramatically reduces its effect on thrombin inactivation mediated by heparin cofactor II. Sulfated fucan with ~45 tetrasaccharide repeating units binds to heparin cofactor II but is unable to link efficiently the plasma inhibitor and thrombin. This last effect requires chains with ~100 or more tetrasaccharide repeating units. The template mechanism may predominate over the allosteric effect in the case of the linear sulfated fucan inactivation of thrombin in the presence of heparin cofactor II. The linear sulfated fucan requires significantly longer chains than mammalian glycosaminoglycans to achieve anticoagulant activity [53].

A low molecular weight fucoidan (LMWF) obtained from *A. nodosum* by acid hydrolysis, whose predominant repeating structure was [→3)-α-l-Fuc(2SO_3_^-^)-(1→4)-α-l-Fuc(2,3diSO_3_^-^)-(1]_n_ and whose Mw was 3,090 Da, has *in vitro* anticoagulant activity, indicating that branched structures were not always necessary for anticoagulant activity. The degree of sulfation of LMWF was three sulfates per disaccharide, which was the same as that of the major repeating unit of heparin, [4)-α-l-IdoA(2 SO_3_^-^)-(1→4)-β-d-Glc-(NSO_3_^-^,6 SO_3_^-^)-1→]_n_ [18].

Some studies showed that sugar composition (fucose, galactose, etc.) of fucoidan may be related to anticoagulant activity [25,48]. But we speculate that it is not the sugars but rather the sulfates on these sugars that effect anticoagulant activity. The results of Pereira *et al.* indicate that a 2-sulfated, 3-linked α-l-galactan, but not an α-l-fucan, is a potent thrombin inhibitor mediated by antithrombin or heparin cofactor II [45, 54, 55]. Uronic acid is not necessary for the anticoagulant activity, but it can enhance the anticoagulant activity through improving the flexibility of the sugar chain [56].

Many fucoidans prolong APTT distinctly, but delay TT little, which suggest that anticoagulant activity mainly came from the endogenesis coagulant approach restrained by fucoidans [56,57]. However, fucoidan from the fermented brown seaweed *Sargassum fulvellum* is able to inhibit both intrinsic and extrinsic blood coagulation pathways [58].

Thrombin acts an important role in thrombosis, so thrombin inhibitor becomes the main content of studies on antithrombotic drugs. Lots of studies show that anticoagulant activity of fucoidan is mainly mediated by thrombin inhibition by heparin cofactor II. It also accelerated thrombin and factor Xa inhibition by antithrombin but at a lower potency [49, 52, 53, 59, 60]. However, Kuznetsova *et al.* reported that anticoagulant properties of fucoidan from *F. vesiculosus* were determined by thrombin inhibition mediated via plasma antithrombin-III *in vitro* and *in vivo* experiments, whose anticoagulant activity was similar to that of heparin [61].

Mourao has summarized the anticoagulant and antithrombotic activities of sulfated fucans. The algal and invertebrate sulfated fucans have potent anticoagulant activity, mediated by antithrombin and/or heparin cofactor II. This aspect was clarified as studies were extended to invertebrate polysaccharides. These definitively established that regular, linear sulfated α-l-fucans and sulfated α-l-galactans express anticoagulant activity, which is not simply a function of charge density, but depends critically on the pattern of sulfation and monosaccharide composition [62].

Melo *et al*. investigated the mechanisms of anticoagulant activity mediated by sulfated galactans. The anticoagulant activity of sulfated polysaccharides is achieved mainly through potentiation of plasma cofactors, which are the natural inhibitors of coagulation proteases. Their results indicated the following: 1) structural requirements for the interaction of sulfated galactans with coagulation inhibitors and their target proteases are not merely a consequence of their charge density; 2) the structural basis of this interaction is complex because it involves naturally heterogeneous polysaccharides but depends on the distribution of sulfate groups and on monosaccharide composition; 3) sulfated galactans require significantly longer chains than heparin to achieve anticoagulant activity; 4) possibly, it is the bulk structure of the sulfated galactan, and not a specific minor component as in heparin, that determines its interaction with antithrombin; 5) sulfated galactans of ~15 to ~45 kDa bind to antithrombin but are unable to link the plasma inhibitor and thrombin. This last effect requires a molecular size above 45 kDa; 6) sulfated galactan and heparin bind to different sites on antithrombin; 7) sulfated galactans are less effective than heparin at promoting antithrombin conformational activation. Overall, these observations indicate that a different mechanism predominates over the conformational activation of antithrombin in ensuring the antithrombin-mediated anticoagulant activity of the sulfated galactans. Possibly, sulfated galactan connects antithrombin and thrombin, holding the protease in an inactive form. The conformational activation of antithrombin and the consequent formation of a covalent complex with thrombin appear to be less important for the anticoagulant activity of sulfated galactan than for heparin. Their results demonstrate that the paradigm of heparin-antithrombin interaction cannot be extended to other sulfated polysaccharides. Each type of polysaccharide may form a particular complex with the plasma inhibitor and the target protease [63].

Fucoidan also express antithrombotic activity when tested on *in vivo* models of venous and arterial thrombosis in experimental animals [62]. A sulfated galactofucan isolated from the brown alga *Spatoglossum schroederi* showed no anticoagulant activity on several *in vitro* assays. Nevertheless, it had a potent antithrombotic activity on an animal model of experimental venous thrombosis. This effect is time-dependent, reaching the maximum 8 h after its administration compared with the more transient action of heparin. The effect was not observed with the desulfated molecule. Furthermore, the sulfated galactofucan was 2-fold more potent than heparin in stimulating the synthesis of an antithrombotic heparan sulfate by endothelial cells. Again, this action was also abolished by desulfation of the polysaccharide [30].

Fucoidans may have a potential application as anticoagulant drugs, antithrombotic drugs or functional food and medicinal biological materials with few side effects. They can also serve as research reagents to investigate and distinguish among a variety of interrelated events, such as coagulation, bleeding, thrombosis and platelet aggregation [56, 62, 64].

### Antivirus activity

In recent years, it has been demonstrated that sulfated polysaccharides (including fucoidan) exhibited antiviral activities both *in vivo* and *in vitro*, of interest in view of their low cytotoxicity compared with other antiviral drugs currently used in clinical medicine.

Fucoidan of *Laminaria japonica* has anti RNA and DNA virus functions. The antivirus effects of fucoidan on infection due to poliovirus III, adenovirus III, ECHO6 virus, coxsackie B3 virus and coxsackie A16 are remarkable. Fucoidan can inhibit the development of cytopathic effect (CPE) and protect cultural cells from infection caused by above viruses [65].

Herpes is an infection that is caused by a herpes simplex virus (HSV). Fucoidans from *Adenocytis utricularis* [27], *Undaria pinnatifida* (Mekabu) [22], *Stoechospermum marginatum* [11], *Undaria* *pinnatifida* [66], *Cystoseira indica* [67] and *Undaria pinnatifida* [68] show antiviral activities against HSV-1 and HSV-2 without cytotoxicity for Vero cell cultures [67]. Furthermore, fucoidans show inhibitory activities against the replication of several enveloped virus such as human immunodeficiency and human cytomegalovirus [27].

Fucoidan has no direct inactivating effect on virions in a virucidal assay. The mechanism of antiviral activities of fucoidan is to inhibit viral sorption so as to inhibit viral-induced syncytium formation [67]. Electron microscopic investigation of tobacco mosaic virus mixed with fucoidan often showed agglutinated virions [69]. Oral intake of the fucoidan might take the protective effects through direct inhibition of viral replication and stimulation of both innate and adaptive immune defense functions [68]. Sulfate is necessary for the antiviral activity [27, 66]. Sulfate located at *C*-4 of (1→3)-linked fucopyranosyl units appears to be very important for the anti-herpetic activity of fucoidan [67].

There was no correlation between the antiviral and anticoagulant properties [65]. Some fucoidans have antivirus activity but no anticoagulant activity [11,67], and others present the both activities [70].

Some fucoidans are present in large quantities in dietary brown seaweed food products, which are eaten frequently in Asian countries. Doh-ura *et al.* reported that fucoidan from popularly eaten brown algae had antiprion activity and delayed disease onset when it was ingested after the enteral prion infection. Dietary seaweed fucoidan delays the onset of disease of enterally infected mice with scrapie when given orally for 6 days after infection, but not when given before the infection. Daily uptake of fucoidan might be prophylactic against prion diseases caused by ingestion of prion-contaminated materials, although further evaluation of its pharmacology remains to be done [71].

### Antitumor and immunomodulatory activity

Antitumor activity of many polysaccharides has been reported in recent years. Fucoidans from *Eisenia bicyclics* and *L. japonica* are effective against sarcoma 180 [72, 73]. Fucoidan of *L. japonica* can inhibit hepatoma QGY7703 cells into logarithmic phase in vitro, accordingly restraining the growth of tumor [74]. Fucoidan was found to inhibit proliferation and induce apoptosis in human lymphoma HS-Sultan cell lines [75]. Fucoidans from *L.* saccharin*a*, *L. digitata, F. serratus, F. distichus* and *F. vesiculosus* strongly blocked MDA-MB-231 breast carcinoma cell adhesion to platelets, an effect which might have critical implications in tumor metastasis [47].

Alekseyenko *et al.* studied the antitumor and antimetastatic activities of fucoidan from *Fucus evanescens* in C57Bl/6 mice with transplanted Lewis lung adenocarcinoma. Fucoidan after single and repeated administration in a dose of 10 mg/kg produced moderate antitumor and antimetastatic effects and potentiated the antimetastatic, but not antitumor activities of cyclophosphamide. Fucoidan in a dose of 25 mg/kg potentiated the toxic effect of cyclophosphamide [76].

Fucoidans inhibit tumour cell adhesion to various substrata, but their mechanisms of action are not fully understood. Based on the study of fucoidan binds to fibronectin, Liu *et al.* hypothesized that fucoidan inhibits the adhesion of MDA-MB-231 cells to fibronectin i) by blocking the protein's heparin- and cell-binding domains, ii) by modulating the reorganization of the integrin alpha5 subunit and iii) by down-regulating the expression of vinculin [77]. Adult T-cell leukemia (ATL) is caused by human T-cell leukemia virus type 1 (HTLV-1) and remains incurable. Fucoidan significantly inhibited the growth of peripheral blood mononuclear cells of ATL patients and HTLV-1-infected T-cell lines but not that of normal peripheral blood mononuclear cells [78]. The animals were fed with a diet containing 1% fucoidan from Mekabu (0.034±0.003 g/mouse/day) for 10 days and subcutaneously (*s. c.*) inoculated with A20 leukemia cells. Thereafter, the mice were fed with the diet containing fucoidan for 40 days. Mekabu fucoidan inhibited tumors by 65.4 % [79].

Fucoidan of *L. japonica* can restore the immune functions of immunosuppressed mice, and it was an immunomodulator acting directly on macrophage and T lymphocyte [80]. It can also promote the recovery of immunologic function in irradiated rats. The mechanism is associated with the arrest of lymphocyte apoptosis by fucoidan [81,82]. Fucoidan can induce the production of interleukin-1 (IL-1) and interferon-γ (IFN-γ) *in vitro*, enhance the functions of T lymphocyte, B cell, macrophage and natural killer cell (NK cell) and promote the primary antibody response to sheep red blood cell (SRBC) *in vivo* [83]. High molecular-weight fucoidan prepared from *Okinawa mozuku* (*Cladosiphon okamuranus*) promotes an increase in the proportion of murine cytotoxic T cells [84]. Fucoidan from *F. vesiculosus* has immunostimulating and maturing effects on dendritic cells (DCs), which are powerful antigen-presenting cells, via a pathway involving at least nuclear factor-κB (NF-κB) [85].

Many polysaccharides obtained from natural sources are considered to be biological response modifiers and have been shown to enhance various immune responses. Choi *et al.* investigated the immunomodulating effects of arabinogalactan and fucoidan *in vitro*. Mouse spleen lymphocytes became cytotoxic to tumor cells after culture with AG and FU at concentrations of 10–100 *µ*g/mL. Also, arabinogalactan and fucoidan were mitogenic in spleen lymphocytes and peripheral macrophages. Macrophages treated with arabinogalactan and fucoidan (10–100 *µ*g/mL) exhibited induced tumoricidal activity and increased phagocytosis, lysosomal enzyme activity, and production of nitrite, H_2_O_2_, tumor necrosis factor (TNF)-*α*, and interleukin (IL)-6. However, arabinogalactan and fucoidan had little effect on the level of IL-1*β*. Thus, the tumoricidal effect of arabinogalactan- and fucoidan-activated macrophages appeared to be mainly mediated by production of free radicals (NO and H_2_O_2_) and cytokines (TNF-*α* and IL-6). These data suggest that arabinogalactan and fucoidan are activators of lymphocytes and macrophages. This property may contribute to their effectiveness in the immunoprevention of cancer [86].

Besides of directly inhibiting the growth of tumor cells, fucoidans can also restrain the development and diffusion of tumor cells through enhancing body’s immunomodulatory activities. Fucoidan can kill the tumor cells directly [74], it has direct anti-cancer effects on human HS-Sultan cells through caspase and ERK pathways [75]. Fucoidan increases the quantity of macrophages [73], and mediates tumor destruction through type 1 T-helper (Th1) cell and NK cell responses [79].

### Antioxidant activity

Lots of studies show that fucoidan presents significant antioxidant activity in experiments *in vitro*. It is an excellent natural antioxidant and has great potential for preventing free radical-mediated diseases. Fucoidan from *L. japonica* can prevent the increase of lipid peroxide (LPO) in serum, liver and spleen of diabetic mice obviously. However, no inhibition effect was found on both spontaneous lipid peroxidation of homogenates and that induced by Cys/FeSO_4_ *in* *vitro* [87]. This fucoidan had strong scavenging effect on superoxide radical, its effect on hydroxyl radical was weak; it had less influence on 1,1-diphenyl-2-picryl-hydrazyl (DPPH). It inhabited H_2_O_2_-induced hemolysis of rat erythrocytes effectively and showed significant protective effect on lipid peroxidation of liver homogenate in rat induced by FeSO_4_-ascorbic acid [88]. Micheline *et al.* reported that fucoidan (homofucan) from *F. vesiculosus* and fucans (heterofucans) from *Padina gymnospora* had an inhibitory effects on the formation of hydroxyl radical and superoxide radical. Fucan showed low antioxidant activity relative to fucoidan [89].

Antioxidant activity relates to the molecular weight and sulfate content of fucoidan. Fucoidan fractions from *L. japonica* had excellent scavenging capacities on superoxide radical and hypochlorous acid, except the highly sulfated fraction L-B. In LDL oxidation system, low molecular weight fractions L-A and L-B exhibited great inhibitory effects on LDL oxidation induced by Cu^2+^, however F-A and F-B had little inhibitory effects in this system due to their large molecular weights. [90]. Both molecular mass and sulfate content of fucoidan played very important roles in the effects on the azo radicals 2-2′-Azobis(2-amidinopropane) dihydrochloride (AAPH) induced LDL oxidation [91]. The correlation between the sulfate content and scavenging superoxide radical ability was positive, the ratio of sulfate content/fucose was an effective indicator to antioxidant activity of the samples [92].

### Reducing blood lipids

Fucoidan is a kind of active materials similar to sialic acid, it can enhance the negative charges of cell surface so as to effect the aggradation of cholesterol in blood, as a result decreasing the cholesterol in serum. Fucoidan of *L. japonica* remarkably decreased total cholesterol, triglyceride and LDL-C, and increase HDL-C in serum of mice with hypercholesterolemia and rats with hyperlipidaemia, and efficiently prevented the formation of experimental hypercholesterolemia in mice [93,94]. It can also remarkably reduce the contents of cholesterol and triglyceride in serum of patients with hyperlipidaemia, without side-effect of damaging liver and kidney [95]. Low molecular sulfated fucan (average Mw=8000 Da) prepared from *L. japonica* can distinctly reduce blood lipids of hyperlipidemic rats [96]. Fucoidan oligosaccharides show good antihypertensive effects on renovascular hypertensive rats and one of the mechanisms underlying the antihypertensive effects might be that they can inhibit the production of plasma angiorensin II [97].

### Anticomplementary activity

The complement system is a major component of the immunity and is mainly involved in the innate and humoral response. It also allows the link between the innate immunity and the adaptive defense. An uncontrolled activation of this system can be harmful for the host organism, as observed in ischemic and anaphylactic shocks or xenograft rejection. Algal fucoidan from *A. nodosum* has been first described as an anticomplementary molecule by Blondin *et al.* [98]. Since this first report, other fucoidans from fucales (*F. evanescens*) and from other brown algae of Laminariale order have been also described as inhibitors of the complement [99]. Tissot *et al.* have reviewed the research progress of anticomplementary activity of fucoidans [100]. 

### Therapeutic potential in surgery

The structural and anionic characteristics of fucoidan are similar to those of heparin. Heparin stimulates production of hepatocyte growth factor (HGF), which has key roles in tissue regeneration. Fucoidan and fucoidan-derived oligosaccharides have similar ability to stimulate production of HGF as heparin and heparin-derived oligosaccharides. This induction of HGF by heparin or fucoidan and their oligosaccharide derivates occurs primarily at the level of translation, probably via the same mechanism. Fucoidan may thus be useful to protect tissues and organs from various injuries and diseases, via mechanisms involving HGF [101].

In recent years, therapeutic angiogenesis has been proposed in the treatment of chronic ischemia. In animals, it was shown that basic fibroblast growth factor (FGF-2), which is mitogenic for vascular endothelial cells, fibroblasts, and smooth muscle cells, can induce angiogenesis *in vivo*. High-molecular-weight (HMW) fucoidans are known to bind growth factors, such as FGFs, and protect them from proteolysis. A fraction of low-molecular-weight (LMW) fucoidan (7±2kDa) was obtained by radical depolymerization of HMW extracts from brown seaweed [102] and was devoid of any direct antithrombin effect [103]. LMW fucoidan can promote therapeutic revascularization, potentiate FGF-2 activity, mobilize stromal-derived factor (SDF)-1, and facilitate angiogenesis in a rat model of critical hindlimb ischemia. This natural compound could be of interest as an alternative for conventional treatment in critical ischemia [104]. The chitosan/fucoidan complex-hydrogel has high affinity for FGF-2. The interaction of FGF-2 with chitosan/fucoidan complex-hydrogel substantially prolonged the biological half-life time of FGF-2. It also protected FGF-2 from inactivation, for example by heat and proteolysis, and enhance FGF-2 activity. The complex-hydrogel can control the release of biologically active FGF-2 molecules through slow diffusion and biodegradation and that subsequent induction of vascularization occurs. For example, when FGF-2-containing complex-hydrogel was subcutaneously injected into the back of mice, significant neovascularization and fibrous tissue formation were induced near the site of injection at 1 week, and the complex-hydrogel was biodegraded and disappeared after 4 weeks. So, FGF-2-containing chitosan/fucoidan micro complex-hydrogel is thus useful and convenient for treatment of ischemic disease [105].

Fucoidan can reduce rat smooth muscle cell (SMC) proliferation *in vitro* in a more intensive manner than heparin [106]. LMW fucoidan with high affinity for SMC and sustained plasma concentration markedly reduces intimal hyperplasia, suggesting its use for the prevention of human in-stent restenosis [107].

Transplant arteriosclerosis is the main cause of long-term failure after cardiac transplantation. Vascular rejection is thought to be due to intimal proliferation occurring in response to arterial wall immune-mediated injury. Low molecular weight fucan (LMWF) treatment appeared very effective in a rat cardiac allograft model to prevent arterial and parenchymal lesions occurring in response to alloimmune injury. However this protective effect does not appear to depend on mobilization of bone marrow-derived cells [108]. LMWF treatment significantly prevented allograft intimal proliferation, permitted a normalization of the intima/media ratio, reduced intimal thickness and induced the presence of an endothelial cell lining in the vascular graft. This may suggest a novel therapeutic strategy in the prevention of transplant arteriosclerosis [108,109].

Aberrant wound healing, either causing scarring or chronic wounds, is a significant cause of morbidity. There is therefore, considerable interest in agents which can modulate certain aspects of the wound healing process. Healing of dermal wounds with macromolecular agents such as natural polymers is one of the research areas of the pharmaceutical biotechnology. Fucoidan has been shown to modulate the effects of a variety of growth factors through mechanisms thought to be similar to the action of heparin. It has properties which may be beneficial in the treatment of wound healing [110]. Fucoidan-chitosan films can promote the re-epithelization and contraction of the wound area. When rabbits with superﬁcial dermal burns was treated with fucoidan-chitosan gel, ﬁbroplasia and scar was observed on wounds after 7 days treatment, the best regenerate dermal papillary formation and the fastest closure of wounds were observed after 14 days treatment. So the fucoidan-chitosan hydrogel formulations can be suitable for the treatment of dermal burns [111,112]. 

### Anti-inflammatory

All fucoidans obtained from nine species of brown algae inhibited leucocyte recruitment in an inflammation model in rats, and neither the content of fucose and sulfate nor other structural features of their polysaccharide backbones significantly affected the efficacy of fucoidans in this model [47]. Mekabu fucoidan can relieve the pulmonary inflammation and down-regulated Th2-dominated responses, which might be useful for treating allergic inflammation [113].

Yang *et al.* evaluated the effect of fucoidan on the expression of inducible nitric oxide synthase (iNOS) in a macrophage cell line, RAW264.7. Low concentration range of fucoidan (10 μg/ml) increased the basal expression level of iNOS in quiescent macrophages. They found for the first time that fucoidan inhibited the release of nitric oxide (NO) in RAW264.7 cells stimulated with lipopolysaccharide (LPS). This inhibitory effect on activator protein 1 (AP-1) activation by fucoidan might be associated with its NO blocking and anti-inflammatory effects [114].

### Gastric protection

Fucoidan from *Cladosiphon okamuranus* tokida is a safe substance with potential for gastric protection [115]. An antiulcer agent and an adhesion inhibitor for Helicobacter pyroli comprising fucoidan as an active ingredient has been provided. The novel drug is effective in treating and preventing of ulcers on the gastric mucosa and in inhibiting the adhesion of Helicobacter pyroli on the gastric [116]. Fucoidan of *Cladosiphon okamuranus* showed growth inhibition of stomach cancer cells but did not show any effects on normal cells. The sulfate content and molecular weight of the fucoidan were 9.8% (w/w) and approximately 3,200,000, respectively [117].

### Against hepatopathy

Fucoidan prevented concanavalin A-induced liver injury by mediating the endogenous interleukin (IL)-10 production and the inhibition of proinflammatory cytokine in mice [118]. The dietary fiber in edible brown seaweeds (*Laminaria sp*., *Sargassum fulvellum* and *Eisenia bicyclis*) had the repressive effect against D-galactosamine (D-GalN)-induced hepatopathy and the protective effect was caused at least in part by fucoidan [119,120]. Hepatic fibrosis results from chronic damage to the liver in conjunction with the progressive accumulation of fibrillar extracellular matrix proteins. There are over 100 million people with hepatic fibrosis in the world. Administration of fucoidan reduced CCl_4_-induced acute and chronic liver failure. Hepatic fibrosis induced by CCl_4_ was also attenuated by injection of fucoidan. Damage to hepatocytes and activation of hepatic stellate cells are key events in liver fibrosis, and, interestingly, treatment of hepatocytes with fucoidan prevented CCl_4_-induced cell death and inhibited the proliferation hepatic stellate cells. So fucoidan might be a promising anti-fibrotic agent possessing dual functions, namely, protection of hepatocytes and inhibition of hepatic stellate cell proliferation [121].

### Against uropathy and renalpathy

Oxalate, one of the major constituents of renal stones, is known to induce free radicals which damage the renal membrane. Damaged epithelia might act as nidi for stone formation aggravating calcium oxalate precipitation during hyperoxaluria. Fucoidan from *F. vesiculosus* can relieve the oxalate-induced free radical injury [122]. Mitochondrial damage is an essential event in hyperoxaluria, and fucoidan was able to effectively prevent it and thereby the renal damage in hyperoxaluria [123]. Fucoidan administration was able to maintain the integrity of erythrocyte membrane and decrease the damage to erythrocytes in hyperoxaluria [124]. Advocation of fucoidan enhanced the antioxidant status, thereby preventing membrane injury and alleviating the microenvironment favorable for stone formation [125]. Urinary supersaturation-induced crystal formation has been attributed as one of the key factor for the pathogenesis/progression of lithogenesis. In hyperoxaluric rats, there was an increased excretion of calcium oxalate monohydrate crystals in urine along with crystal deposition in renal tissues; this was prevented by fucoidan treatment [126].

Fucoidan may ameliorate epithelial–mesenchymal transition and renal ﬁbrosis in adenine-induced chronic renal failure rats [127]. The elevated urinary protein excretion and plasma creatinine due to the induction of Heymann nephritis were significantly reduced by fucoidan from *L. japonica* by oral intubation. This indicated that fucoidan has a renoprotective effect on active Heymann nephritis and is a promising therapeutic agent for nephritis [128].

## Conclusions

The fucoidans of brown algae are complex and heterogeneous, and their refined structures have not been very clear until now. However, their biological activities are so attractive that much research is being done on their structures and bioactivities every year. Because most biological activity studies are carried out using a relatively crude fucoidan preparation, it is not easy at present to determine the relationships between activity and structure. But it has become clear that at least some of these activities are not merely an effect of high charge density but have distinct structural specificities. Future conformational studies of well-defined fucan structures should lead to better understanding of the biological properties of fucoidans.

Brown algae are abundant in the world is and some species (*H. fusiforme*, *L*. *japonica*, etc.) have been cultivated in a large scale. Now most brown algae are consumed as foods or food additives, while some of them have been developed into new drugs and functional foods. Through chemical modification, for example sulfation at special positions, some fucoidans’ bioactivities were improved remarkably. Deeply studying the structure of fucoidans and exploring the relationship activity and structure can provide theory foundation for developing and utilizing the brown algae resource.
